# Do young adults see value in advance directives?

**DOI:** 10.1093/geroni/igab046.3087

**Published:** 2021-12-17

**Authors:** Arianna Stone, Yuchi Young, Taylor Perre, Kuo-Piao Chung, Ya-Mei Chen

**Affiliations:** 1 University at Albany School of Public Health , Albany , New York, United States; 2 State University of New York at Albany, Rensselaer, New York, United States; 3 University at Albany, Rensselaer, New York, United States; 4 National Taiwan University, School of Public Health, Taipei, Taipei, Taiwan (Republic of China); 5 National Taiwan University, Taipei, Taipei, Taiwan (Republic of China)

## Abstract

Many Americans avoid end-of-life care planning; only 26% have completed an advance directive (AD). An AD promotes end-of-life care with dignity allowing individuals to make end-of-life treatment and care decisions before they are unable to do so. Previous studies related to ADs are focused on older adults with serious illness or people with functional/mental disability. The objective of this survey is to better understand young adults' knowledge of and attitude toward ADs and their preferences for ADs related to treatment and care options. Methods. Participants include graduate students (n=25) attending a state university in New York State (NYS). Data were collected using two ADs (Five Wishes; Medical Orders for Life-Sustaining Treatment (MOLST)) and one survey questionnaire. Summary statistics and multivariate models will be used to address the study aims. Results. Preliminary results show the average age was 23 years, 72% were female, 48% White, and 44% Black. The majority of young adults hadn’t completed an AD; however, their attitude toward ADs was positive; the majority believe it is important to have an AD prepared at their current age; and they believe young adults would willing to fill out ADs. Young adults can make difficult treatment and care decisions when the situation requires it. Conclusion. The study findings can be useful to policy makers, healthcare providers and other stakeholders in promoting population-based healthcare decision-making. Limitation. Participants were recruited from one university in NYS; thus, the study results may be generalized to a population sharing similar characteristics.

